# The Gift of Observation: An Interview with Mary Lyon

**DOI:** 10.1371/journal.pgen.1000813

**Published:** 2010-01-22

**Authors:** Jane Gitschier

**Affiliations:** Department of Medicine and Pediatrics, University of California San Francisco, San Francisco, California, United States of America

For more than 60 years, Mary Lyon has had an intimate relationship with the house mouse. She has devoted herself to the discovery and description of a wide variety of mutants, arguably as prolific as anyone in the field. She co-edited the mouse bible “Genetic Variants and Strains of the Laboratory Mouse” and untangled the knots in the *t*-complex. And, in a link with posterity, her last name now forms the basis for a word—“lyonization”—synonymous with the mammalian random X-inactivation process that she first hypothesized a half-century ago.

I was keen to interview Mary but hesitant, as I knew she had retired. Thanks go to my fellow *PLoS Genetics* editor, Elizabeth [Lizzy] Fisher, who had done graduate work with Lyon and encouraged me to email her. While no longer running a lab, Lyon still comes to work a few days a week at the Medical Research Council (MRC) Unit at Harwell in the United Kingdom, and she agreed to meet with me there.

**Image 1:**
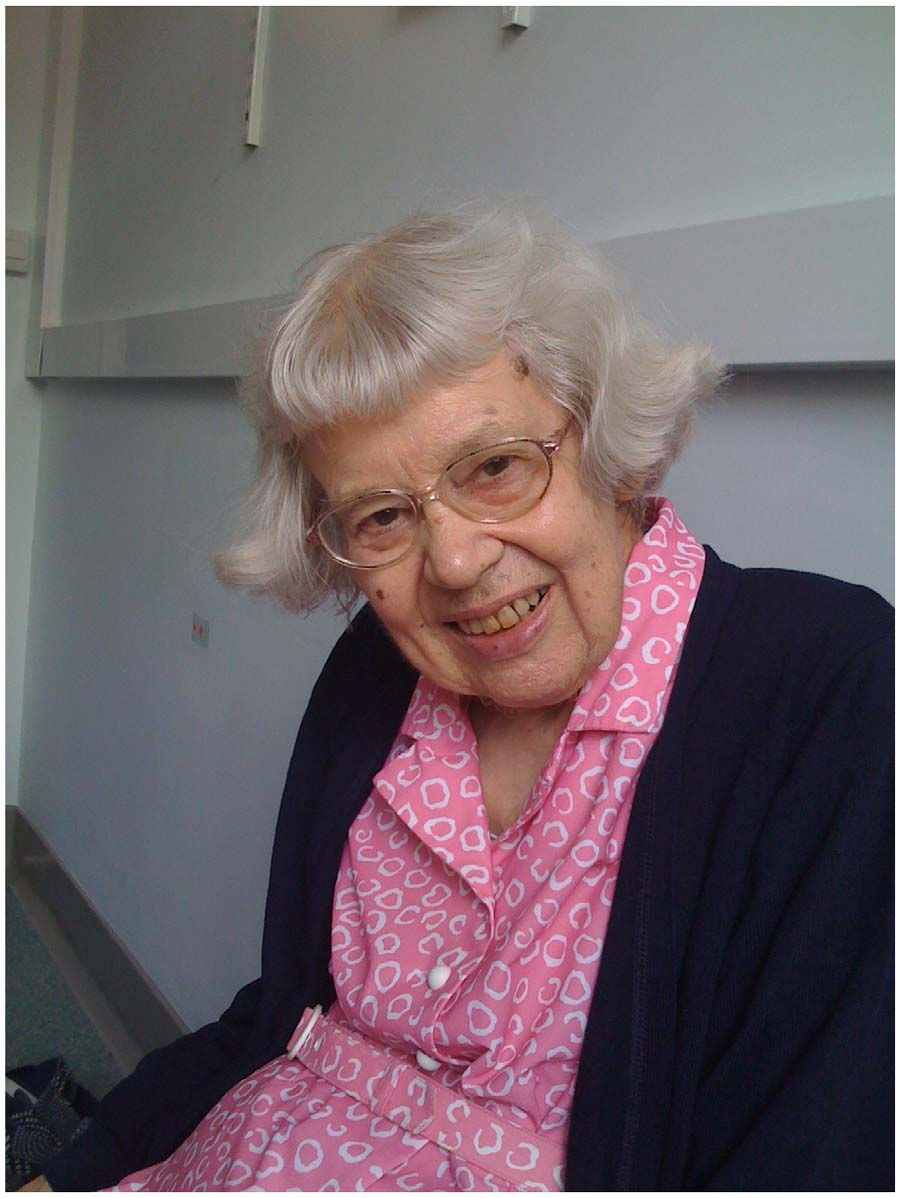
Mary Lyon.

En route to see Mary (Image 1), as she seems to be universally and reverentially referenced, I found Harwell itself a study in contrasts. The facility surprised me in its starkness, its aging buildings and dandelion-bespeckled grass surrounded by a chain-link fence, apparently in response to the potential threat by animal rights activists. I struck up a conversation with the guard, who opined that Mary has been unfairly denied a knighthood, not only because she is a woman, but also, perhaps, because work involving animals is politically charged. I then wended my way toward the meeting room and was ushered in. The door opened to the warmth of Mary standing there, wooden cane in hand, radiating a smile, and quietly waiting to offer me a beverage. Despite her soft voice, I knew I was in the presence of a giant.

**Lyon:** Would you like a cup of tea or coffee?

**Gitschier:** Yes. Would you? Tea?

I want to thank you for agreeing to be interviewed. I'm interested first in your upbringing and what got you interested in science.

**Lyon:** I was the eldest of three children. My father was a civil servant; my mother was a school teacher when she was young.

My family lived in several places. I was born in Norwich, and then my parents moved to Yorkshire when I was four to six, then to Birmingham when I was 10 and then to Woking in Surrey when I was 14.

The grammar school I attended in Birmingham was a very good school. I got interested in science there. At first I was interested in physics and chemistry but then I quickly changed to biology. I won a prize in an essay competition when I was about age ten, and the prizes were four books on nature study. And that got me interested in biology.

**Gitschier:** What about your brother and sister? Were they also interested in science?

**Lyon:** No, they weren't. My brother became an accountant. My sister first worked as a school teacher and then as a social worker.

But the person who was interested in science is my father's sister's son, Kenneth Blaxter. He was an expert on farm animal nutrition. He was the director of the Rowett Nutrition Research Laboratory in Scotland. He won prizes and he was knighted and so on.

**Gitschier:** For university, you chose to attend Cambridge. Was it very common for women to be in Cambridge at the time?

**Lyon:** No. At that time women were not members of the University. There were two colleges for women. I was in Girton and the other was Newnham, but the women were in the minority because they were restricted to these two colleges. The men restricted us to 500 women, and there were more than 5,000 men. We used to go to the lectures with men, took the same practical courses as the men, and took the same exams as the men, but, officially, we didn't get a degree. We got a “titular” degree.

**Gitschier:** Really? When did you graduate?

**Lyon:** I graduated in 1946. And of course, the Second World War changed the position of women in the world. And in 1948, Cambridge admitted women [officially] to the University.

**Gitschier:** It must have been very unusual for women to go on to do a Ph.D.

**Lyon:** Yes, it was. I was in a women's college, of course, and several women went on to a Ph.D.

**Gitschier:** What did your parents think about your choice to continue with a Ph.D? Were they supportive?

**Lyon:** Yes, I think so. They wanted me to get married at one point.

**Gitschier:** What did you think about that idea?

**Lyon:** I didn't like it.

**Gitschier:** Was there someone in particular they wanted you to marry?

**Lyon:** No.

**Gitschier:** Just in principle, then! So, because of the war, there seemed to be more educational opportunities for you.

**Lyon:** Yes, there were. I didn't really realize how much more opportunity there was, but there certainly was at the time. This was because during the war, the government restricted very much the men who could go to university.

Medical students could go to university and men doing physics and chemistry, because physics and chemistry were needed in the war effort. But in zoology there were very few men. The government did allow a few men to do things like zoology and botany if they were really good, believing that if they got high marks on their exams they would do some research that would help the war effort.

But there were fewer men in relation to the number of women at that time because they were called up to the military.

**Gitschier:** When you graduated in 1946, [C.H.] Waddington wasn't there, but you had wanted to work with him. You ended up with [R.A.] Fisher instead. What was he like?

**Lyon:** Fisher was a very brilliant man but a very eccentric man. He was difficult to work with. He was brilliant in a logical mathematical sense. So we learned about ratios of normal and affected offspring, that sort of thing.

**Gitschier:** How old was Fisher when you worked with him?

**Lyon:** In his 50s.

**Gitschier:** Had he worked with mice for a very long time?

**Lyon:** Yes, I think so. He was appointed Professor of Genetics in Cambridge round about the time I came to Cambridge in 1943. Before that he was in the Rothamsted plant research station in the outskirts of London. In Cambridge he worked on both plants and mice.

**Gitschier:** And did he have other graduate students besides you?

**Lyon:** Sir Walter Bodmer was a student of his [post-Lyon]. Anthony Edwards was also a student, a contemporary of Walter Bodmer, I think. Various people went to work at the Cambridge lab: Douglas Falconer, Toby Carter. But Fisher didn't get along very easily with people, and he threw out most of them.

**Gitschier:** But he didn't throw you out!

**Lyon:** Well, I felt that I didn't have enough facilities to do my Ph.D. there. I was trying to do dissection of mice and to breed mice and needed facilities for histology. So I moved to Edinburgh, which is where Waddington was, in Genetics. And there were facilities for doing mouse genetics. Douglas Falconer was my supervisor there in Edinburgh.

**Gitschier:** I see. So, you all jumped ship! What became of poor Fisher?

**Lyon:** He did have people who worked with him for short times and there were one or two people who did get on with him and stayed there. These included Margaret Wallace and George Owen. But Fisher stayed there until he was retiring age.

**Gitschier:** So you started to work on the *pallid* mutant when you were still with Fisher and continued on with that project for your thesis. When did you finish your Ph.D.?

**Lyon:** 1950.

**Gitschier:** At that point what was happening?

**Lyon:** Waddington was very good about getting money for young people to stay on at Edinburgh. He sent in an application to the MRC for a project for me to do. He didn't think of me working for the ARC [Agricultural Research Council] because they weren't giving equal pay for women at the time. He got the MRC money, and that's how I started my post-doc in Edinburgh.

**Gitschier:** And what did you work on?

**Lyon:** I continued to work on *pallid*, but I also worked on the mutagenic effects of radiation, part of a bigger project that Waddington got the money to work on, namely, the mutagenic effect. At that time, after the Second World War and the atomic bombs in Japan, there was a lot of concern about the harmful effects of fallout in the atmosphere. So I was part of this project, which also included studying the actual mutants that we had obtained.

**Gitschier:** What kind of mutants?

**Lyon:** I will just mention a few examples. One was called *ataxia*, a mutant of the nervous system that caused the mouse to have problems walking. There was also *twirler*, a mutant that affected the inner ear of mice—they ran around in circles and had no sense of balance and shook their heads. There were *short-eared* mutants and a type of vitamin-D resistant rickets.

**Gitschier:** A wide spectrum of things!

**Lyon:** The [mutagenesis] project there got us all scared. The head person responsible for the experiment, Toby Carter, said that he couldn't do the mutagenesis experiment without a lot more breeding cages for the mice. And there was no possibility of getting these extra facilities in Edinburgh. Toby was in contact with John Loutit, who was the director of this unit here [in Harwell].

**Gitschier:** So Harwell already existed.

**Lyon:** The MRC had this project at Harwell to study the harmful effects of radiation.

**Gitschier:** So, similar to the Edinburgh project.

**Lyon:** Yes, but they were not doing genetics, they were studying cancer [in mice].

**Gitschier:** There is another woman whose name is on a lot of the papers with you at this time—Rita Phillips. Who is she?

**Lyon:** She was employed as a research assistant in Edinburgh before I was there. And she came to Harwell, too. We moved in 1955.

**Gitschier:** So did you continue to be a post-doc upon your move to Harwell?

**Lyon:** No. People didn't talk about post-docs in those days. People were scientists. You could have a short contract or you could have tenure.

**Gitschier:** So you were a scientist, presumably with a short contract. Renewable?

**Lyon:** Yes. First I had a 3-year contract, then a 5-year contract, then tenure.

**Gitschier:** I want to talk to you about the new X-linked mutants, such as *Tabby* and *mottled*, that were starting to be identified. Where were they discovered?

**Lyon:** They were discovered in Edinburgh by people working with Douglas Falconer.

**Gitschier:** So, even before you moved here, you knew about these mutants.

**Lyon:** Yes, it was a very exciting thing to talk about in those days. No one had found a sex-linked mutant in mouse until then. But we didn't pursue it initially.

**Gitschier:** Did the mutants move to Harwell too?

**Lyon:** Yes, there were quite a lot of different *mottled* mutants early on, and we didn't have all of them. There aren't so many *tabby* mutants, but we did have *Tabby*.

**Gitschier:** When did you first start having this idea about the X chromosome inactivating?

**Lyon:** I was still studying the mutants that we had found in mutagenesis experiments. We found quite a number of *mottled* [mutants], and they weren't all the same. In some the affected males die as embryos; in others they are born and have white coats. The females were variegated. And I found one in which the original animal of this particular mutant was a mottled male, which was odd because males have got only one X chromosome. So why was he mottled?

So we bred from it to find whether the mottled pattern was inherited. This mouse had some daughters who looked like himself and he also had normal daughters and normal sons. So his mottled appearance was inherited. When we bred from his affected daughters, they bred as the previous *mottled* mutants that had been found. That is they had mottled daughters, like themselves, and also affected males, which died. So the females were behaving like ordinary *mottled* mice with a mutant gene on their X chromosome.

But we still had the question of the original mottled male mouse. How did he get to be mottled? Then it occurred to me that he had a mutation that had occurred in him, when he was just an embryo, when he was just a few cells, and that gave rise to one progeny group of cells with a mutant X chromosome and another group of cells with the unmutated, normal X chromosome. So this original mutant male was a mosaic of two types of cells, some with the mutated X chromosome and others with the normal X chromosome.

So then, it occurred to me that if that explanation of him having two types of cells applied to his pattern, could it not also apply to the pattern of his daughters? His daughters could have two types of cells, one with the mutant gene active and one with the normal gene active.

And that involved me in finding out about recent work on the mammalian X chromosome. One important point was that XO mice are normal fertile females, and thus a female mouse needs only one X chromosome for normal development. Furthermore, female mammals have the sex chromatin in their nuclei, and, just recently before that time, Ohno had found that the sex chromatin consisted of one highly condensed X chromosome.

So the female mouse only needs one X chromosome, and in female mice the X chromosome behaves strangely. So I put all those things together and came up with the idea of X-chromosome inactivation.

**Gitschier:** Before you read the literature and pieced all this together, did you already have the idea that in females only one X was active?

**Lyon:** Yes.

**Gitschier:** These mice that you are referring to: were they also the product of radiation?

**Lyon:** No, the original male was a spontaneous mutant.

**Gitschier:** Do you remember the year that original male appeared?

**Lyon:** 1959 or 1960.

**Gitschier:** You published your paper in 1961, so the pieces of the puzzle must have very quickly fallen into place. And do I take it that X-inactivation is also playing a role in the *Tabby* mutant?

**Lyon:** The striped pattern in *Tabby* females is indeed due to X-inactivation. It is not due to differences in pigmentation of the coat but to differences in hair texture. *Tabby* males have an obviously abnormal coat, which looks too sleek. Females have patches of this abnormal hair and where the patches of mutant and normal hair meet, one sees a stripe. The sizes and shapes of patches and stripes in heterozygotes for different X-linked genes depend on the way that the cells underlying the patches migrate and mingle during development.

An interesting example concerns the tortoiseshell cat. The pattern is produced by cells giving black or yellow pigment. If the cat has an autosomal gene for white spotting, patches of black and yellow are larger. This is because the spotting gene reduces the number of pigment cells and hence each precursor cell must cover a wider area and hence produces a larger patch.

**Gitschier:** Lizzy Fisher mentioned to me that one person in particular, Hans Gruneberg, gave you a lot of grief about your hypothesis. Would you like to comment on that and whether that was difficult for you?

**Lyon:** Gruneberg did indeed make things difficult in the early days of X-inactivation. He seemed to have two main objections. Firstly he seemed to think that I was not sufficiently established or eminent enough to put forward such a major idea. Secondly he seemed to have problems with the points mentioned above on sizes and shapes of stripes and patches. The theory does not require that each stripe or patch be derived from a single precursor cell. The *tabby* gene in the mouse provides an example. The gene affects the development of the teeth. If each tooth were derived from a single precursor cell, then each one would be either fully mutant in phenotype or fully normal. In fact, each tooth is intermediate in appearance. This is consistent with the origin of each tooth formed by a small pool of precursors in which some cells have the mutant gene and others the normal gene active. Individual teeth will vary in the proportion of precursor cells with the mutant gene active. Gruneberg seemed to find this difficult. His objections made it difficult to study the stripes and patches of heterozygotes, which were an important source of information in the early days before molecular methods were available.

**Gitschier:** You have now become interested in a new hypothesis, that LINE elements on the X chromosome can serve as a means of transmitting X inactivation in *cis*. That they are somehow boosters. How did you come up with this hypothesis, and are you alone in this theory?

**Lyon:** I thought of this a long time ago because some of the early work on the mouse X chromosome involved X-autosome translocations. And the autosomal part of the translocation does not get inactivated as efficiently as the X chromosome does. Similar evidence from other translocations suggested to me that X-inactivation travels less well in autosomes than it does in X-chromosome material.

So how could that happen? What could there be in X chromosomes that facilitates the spread? I thought it would be something promoting the spread in X chromosomes, rather than inhibiting the spread in autosomes. What could it be? There is a limit to what it could be.

**Gitschier:** What were some of the things you ruled out?

**Lyon:** *Drosophila* have the *roX* genes that work in dosage compensation. But in mammals no one had ever found anything like that. Mammals had not evolved that kind of gene. So what could it be that served as a boosting agent?

And I thought of repetitive elements as booster elements several years before the LINE hypothesis came out. People have found that the X chromosome of the human, and I think also the mouse, is particularly rich in LINE elements, compared to the autosomes. So I thought it could be repetitive elements, particularly the LINE elements.

Since then, there are even more data in the literature to support this, data that come from the human genome sequencing project. The human X chromosome is very rich in LINE elements, particularly in regions where most genes are inactivated, whereas the regions of the X where inactivation does not occur very efficiently are not rich in LINE elements.

But there are other bits of evidence that have not supported the LINE idea terribly well.

**Gitschier:** What kind of evidence?

**Lyon:** There are some odd animals, odd species, that have different types of X inactivation and weird types of DNA, in which LINE elements are not terribly active—not alive—not transcribed. There are some species of vertebrates that have no active LINE elements, but that have X inactivation.

**Gitschier:** What kind of species?

**Lyon:** Particular species of wild mice and rats.

**Gitschier:** Well, is the fact that they be actively transcribed a necessary part of your hypothesis?

**Lyon:** No, I don't think it is.

**Gitschier:** Well then, mechanistically, what could it be about the LINE elements that could make them boosters?

**Lyon:** That is still to be found out.

**Gitschier:** It will be interesting to watch this story evolve.

You wrote a personal history of a half century of mouse work in which you comment that this is just the “hors d'oeuvres and the feast is yet to come.” So I'm wondering if you were to be able to start all over today, is there a project you would choose to work on? I assume you would still choose to be a mouse geneticist!

**Lyon:** I think so, yes. It would be nice to work on the genetics of behavior. This is an area that will be interesting to work out.

**Gitschier:** Did you feel a life in research was a good fit for you?

**Lyon:** I think so, yes. Teasing out problems and applying the scientific method to problems. The thing I didn't like about it when I got to retirement age was how much admin there is: staff appraisals, annual reports, project costings. And there is a lot of admin to do with animal experiments in this country.

**Gitschier:** When did you retire?

**Lyon:** 1990.

**Gitschier:** Were you required to retire [because of age]?

**Lyon:** Yes.

**Gitschier:** Do you have a cat?

**Lyon:** Yes!

**Gitschier:** Me too! What's your cat's name?

**Lyon:** Cindy.

**Gitschier:** And you have a building named after you now. Was that a surprise?

**Lyon:** Yes it was!

**Gitschier:** When you look back on your scientific career, what did you enjoy the most?

**Lyon:** The time I spent in Edinburgh, I would say. It was a very lively academic atmosphere. Leaving and coming here wasn't very good, because we left a big genetics lab and a lot of able and enthusiastic geneticists. Here, there were hardly any other geneticists, and the people weren't as enthusiastic. [But] things did improve here.

**Gitschier:** Are there any other topics you would like to talk about? As long as it's not the *t*-complex, I'm OK.

**Lyon:** People always say about the *t*-complex that they can't understand it. But it seems very sensible to me.

**Gitschier:** I think I'd have to warm up to it. Perhaps some more tea?

